# Cancers of unknown primary diagnosed during hospitalization: a population-based study

**DOI:** 10.1186/s12885-017-3083-1

**Published:** 2017-01-31

**Authors:** William Jones, Gwen Allardice, Iona Scott, Karin Oien, David Brewster, David S. Morrison

**Affiliations:** 1 University of Glasgow, 1 Lilybank Gardens, Glasgow, G12 8RZ UK; 2 NHS Greater Glasgow and Clyde, Glasgow, UK; 3 Scottish Cancer Registry, Edinburgh, UK

**Keywords:** Occult primary neoplasms, Unknown primary tumor

## Abstract

**Background:**

Cancers of Unknown Primary (CUP) are the 3-4^th^ most common causes of cancer death and recent clinical guidelines recommend that patients should be directed to a team dedicated to their care. Our aim was to inform the care of patients diagnosed with CUP during hospital admission.

**Methods:**

Descriptive study using hospital admissions (Scottish Morbidity Record 01) linked to cancer registrations (ICD-10 C77-80) and death records from 1998 to 2011 in West of Scotland, UK (population 2.4 m). Cox proportional hazards models were used to assess effects of baseline variables on survival.

**Results:**

Seven thousand five hundred ninety nine patients were diagnosed with CUP over the study period, 54.4% female, 67.4% aged ≥ 70 years, 36.7% from the most deprived socio-economic quintile. 71% of all diagnoses were made during a hospital admission, among which 88.6% were emergency presentations and the majority (56.3%) were admitted to general medicine. Median length of stay was 15 days and median survival after admission 33 days. Non-specific morphology, emergency admission, age over 60 years, male sex and admission to geriatric medicine were all associated with poorer survival in adjusted analysis.

**Conclusions:**

Patients with a diagnosis of CUP are usually diagnosed during unplanned hospital admissions and have very poor survival. To ensure that patients with CUP are quickly identified and directed to optimal care, increased surveillance and rapid referral pathways will be required.

## Background

Cancers of unknown primary (CUP) are metastatic malignancies for which a primary site has not been identified [1]. They are therefore a disparate group of cancers that present at an advanced stage. The incidence of CUP has risen and then fallen over the past 50 years, [2–5] possibly driven initially by greater diagnostic sensitivity detecting more metastatic disease and then latterly by better identification of the primary site reducing the number of unknown primaries. Worldwide, CUP are the 6^th^ to 8^th^ most common cancers, accounting for 2.3–5% of all cancer diagnoses but the 3^rd^ to 4^th^ most common cause of death from cancer [6, 7]. They comprised 3.2% of all invasive cancers in Scotland between 2001 and 2010 [7]. They were responsible for 7% of United Kingdom (UK) cancer deaths in 2009 [8]. Survival is poor, with a median of 5.6 weeks [7]. Survival is more favourable among patients whose disease mimics clinicopathological features of known metastatic cancers and responds to appropriate treatment (median survival 24 months) but the majority of patients (80–85%) respond poorly to treatment [6].

In the United Kingdom, The National Institute for Health and Care Excellence (NICE) issued a guideline on CUP in 2010 [1]. Its recommendations included that every hospital with a cancer centre should have a dedicated CUP team and that investigations and management be the responsibility of that team. The guideline is currently on NICE’s static list, indicating that no new evidence has become available. The European Society for Medical Oncology (ESMO) issued clinical practice guidelines the following year, emphasising the need to identify those patients with more favourable prognoses so that they might benefit if directed to appropriate multidisciplinary care [9]. The National Cancer Intelligence Network reported on routes to diagnosis of CUP between 2006 and 2010 [10]. They reported that diagnoses were 20% higher among females, nearly 40% were over 80 years old and 57% were diagnosed after an emergency hospital admission. However, there is a paucity of evidence on patterns of hospital care for patients with CUP and therefore on the capacity to better identify patients so that they might be directed to specialist teams.

In order to inform the care of patients diagnosed with CUP during hospital admission, we carried out a descriptive study of all patients diagnosed over the most recent 14-year period for which data were available, using cancer registry data individually linked to hospital and death records.

## Methods

### Study population

The West of Scotland comprises approximately 2.4 million residents, or just over half of the population of Scotland. All residents can access National Health Service hospitals, which are funded through general taxation and care is free at the point of use. There were about 10 acute hospitals in the region over the period of this study with some opening and some closing. The region includes both urban and rural areas, with Glasgow being the largest city (population approx 600,000). All registrations for Cancer of Unknown Primary (International Classification of Diseases (ICD) Revision 10 codes C77-C80) for the region were extracted from the Scottish Cancer Registry for the period January 1998 to December 2011 inclusive. Scottish cancer registrations (Scottish Morbidity Record (SMR) number 6, SMR06) are routinely linked to each individual’s acute hospital records (SMR01) and death records; we obtained all such linked records. The date range was chosen because of enhanced information available from SMR01 from 1998 onwards. ICD-O (International Classification of Diseases for Oncology) morphology codes were grouped into four categories: not otherwise specified (NOS) M8000/0-8046/6, squamous cell carcinoma (SCC) M8050/0-8084/6, adenocarcinoma M8140/0-8420/6 and “other” comprising all remaining morphological types. ICD-O revisions 2 and 3 morphology classifications were used for their appropriate time periods [11].

Socio-economic characteristics of patients were assigned using the Scottish Index of Multiple Deprivation (SIMD) [12]. This uses seven domains of deprivation (income, employment, education, health, access to services, crime and housing) to rank small geographic areas in Scotland from least deprived to most deprived. These have been grouped into national quintiles from 1 (most deprived) to 5 (least deprived).

### Statistical methods

#### Survival analysis

Survival analysis was performed on all incident cases from 1998 to 2011 diagnosed during hospital admission with survival time defined from diagnosis to death from any cause. A censor date of 31^st^ March 2013, the most recent time-point for which complete vital status data was available, was applied. Univariate survival analyses were carried out using log rank tests and the assumption of proportionality assessed by proportionality tests and log-minus-log plots. For multivariate Cox proportional hazards models, we used a forward stepwise approach, entering demographic variables (age, sex) and then all other variables found to be significant in log rank tests. Statistical analysis was performed using Stata v13 (STATA Corp, College Station, TX, USA).

## Results

7,599 diagnoses of CUP were made in the West of Scotland between 1998 and 2011. Table 1 summarises the demographics, ICD-10 site and morphology classifications of these diagnoses. Median age at incidence was 73 years in males and 77 years in females. Patients aged over 70 years accounted for 67.4% of incident cases. The crude rate of diagnoses averaged 18.14 per 100,000 persons between 2008 & 2010.

**Table 1:** Cancer of Unknown Primary patient characteristics, 1998–2011, West of Scotland. *N* = 7599

	1998–2002		2003–2007		2008–2011		Total	
	*N*	Col %	*N*	Col %	*N*	Col %	*N*	Col %
Total	3155	(100)	2669	(100)	1775	(100)	7599	(100)
Sex								
Male	1480	(46.9)	1219	(45.7)	768	(43.3)	3467	(45.6)
Female	1675	(53.1)	1450	(54.3)	1007	(56.7)	4132	(54.4)
	3155	(100)	2669	(100)	1775	(100)	7599	(100)
Age_group								
< 60 year	434	(13.8)	313	(11.7)	244	(13.7)	991)	(13.0)
60–69 years	647	(20.5)	507	(19.0)	328	(18.5)	1482	(19.5)
70–79 years	1124	(35.6)	899	(33.7)	569	(32.1)	2592	(34.1)
> =80 year	950	(30.1)	950	(35.6)	634	(35.7)	2534	(33.3)
SIMD								
Most deprived	1170	(37.1)	976	(36.6)	643	(36.2)	2789	(36.7)
2	800	(25.4)	655	(24.5)	406	(22.9)	1861	(24.5)
3	462	(14.6)	428	(16)	304	(17.1)	1194	(15.7)
4	401	(12.7)	291	(10.9)	195	(11.0)	887	(11.7)
Least deprived	322	(10.2)	319	(12.0)	227	(12.8)	868	(11.4)
ICD10_site								
C77	149	(4.7)	128	(4.8)	117	(6.6)	394	(5.2)
C78	1566	(49.6)	1163	(43.6)	661	(37.2)	3390	(44.6)
C79	608	(19.3)	433	(16.2)	241	(13.6)	1282	(16.9)
C80	832	(26.4)	945	(35.4)	756	(42.6)	2533	(33.3)
Morphology								
Adenocarcinoma	880	(27.9)	615	(23.0)	390	(22.0)	1885	(24.8)
SCC	122	(3.9)	113	(4.2)	95	(5.4)	330	(4.3)
NoS	2039	(64.6)	1853	(69.4)	1201	(67.7)	5093	(67.0)
Other	114	(3.6)	88	(3.3)	89	(5)	291	(3.8)

*N* number of patients, *Col %* column percentage, *SIMD* Scottish Index of Multiple Deprivation, *ICD10_site* International Classification of Diseases, 10^th^ Revision, anatomical site, *SCC* squamous cell carcinoma, *NoS* not otherwise specified, *C77* secondary and unspecified malignant neoplasm of lymph nodes, *C78* secondary malignant neoplasm of respiratory and digestive organs, *C79* secondary malignant neoplasm of other sites, *C80* malignant neoplasm without specification of site

A socioeconomic gradient was present, with 36.7% of cases from the most deprived SIMD quintile versus 11.4% of cases from the least deprived quintile. The most common ICD-10 site codes were C78 (secondary malignant neoplasm of respiratory and digestive organs) at 44.6% and C80 (malignant neoplasm, without specification of site) at 33.3%.

Not otherwise specified (NOS) morphology accounted for 67% of all incidences, adenocarcinomas for 24.8%, SCC for 4.3% and all others 3.8%. Morphology varied by age. Increasing age was associated with increased NOS morphology, comprising 82.2% in patients aged 80 years and older (*Χ* ^2^ = 706, df = 9, *p* <0.001). SCC, adenocarcinoma and other morphologies had greatest proportions in under-60 year-olds, their proportions decreasing with ascending age.

### Patterns of hospital admission

Between 1998 and 2011, 5,397 incident cases of CUP (71% of all CUP diagnoses in the period) were diagnosed during hospital admission. Table 2 summarises patterns of admission. Numbers declined over time, falling by an annual mean of 29.6% between 1998–2002 and 2008–11.

**Table 2:** Hospital admission characteristics and length of stay. Cancer of Unknown Primary diagnosed during hospital admission, 1998–2011, West of Scotland, N = 5397. p for Pearson’s chi-square test

	1998–2002		2003–2007		2008–2011		Total		*p*
	*N*	Col %	*N*	Col %	*N*	Col %	*N*	Col %	
Total	2212	(100)	1939	(100)	1246	(100)	5397	(100)	
Type of admission									
Elective	299	(13.5)	201	(10.4)	117	(9.4)	617	(11.4)	
Emergency	1913	(86.5)	1738	(89.6)	1129	(90.6)	4780	(88.6)	<0.001
Specialty of admission									
Geriatric medicine	258	(11.7)	222	(11.4)	124	(10)	604	(11.2)	
Medicine	1203	(54.4)	1083	(55.9)	751	(60.3)	3037	(56.3)	
Surgery	685	(31.0)	588	(30.3)	329	(26.4)	1602	(29.7)	
Other	66	(3.0)	46	(2.4)	42	(3.4)	154	(2.9)	0.013
days in hospital									
1–7 days	500	(22.0)	450	(22.7)	340	(26.4)	1290	(23.3)	
8–14 days	548	(24.1)	429	(21.6)	284	(22)	1261	(22.7)	
15–30 days	701	(30.9)	618	(31.1)	362	(28.1)	1681	(30.3)	
31+ days	463	(20.4)	442	(22.3)	260	(20.2)	1165	(21.0)	0.016

*N* number of patients, *Col %* column percentage

Most patients (88.6%) were admitted as emergencies. Emergency admissions were older than elective admissions (median ages 76 and 73 years, respectively, Wilcoxon rank-sum *p* <0.001). Of patients >80 years, 91.7% were admitted as emergencies compared to 81.3% of those aged < 60 years (Pearson *χ* ^2^ 76.9363, *p* <0.001). The proportion of emergency admissions increased over time from 86.5% in 1998–2002–90.6% in 2008–2011 (*χ* ^2^ 17.4819, *p* <0.001). The proportions of patients admitted to general medicine increased over time from 54.4% in 1998–2002–60.3% in 2008–2011 (*χ* ^2^ 16.1543, *p* = 0.013). Half of patients (49.3%) died during their hospital stay; this was higher (52.5%) for those who had an emergency admission (p < 0.001). The median length of stay was 15 days, with 21% of patients having a hospital stay of 31 days or more.

### Survival analysis

All incident cases of CUP diagnosed during hospital admission were included in survival analysis. Median survival was 33 days. One year survival was 6.2%; five year survival was 1.4%.

Unadjusted survival analysis showed progressively poorer outcomes with increasing age, such that hazards of death at ≥ 80 years were twice that of those aged under 60 (HR 1.94, 95% CI 1.77 to 2.14) – Table 3.

**Table 3:** Survival of Cancer of Unknown Primary patients by demographic and clinical characteristics. Patients diagnosed during hospital admission, 1998–2011, West of Scotland, N = 5397

	median survival (days)	HR (95% confidence interval)	*P*-value
Gender			
Male	32	1	
Female	34	0.95 (0.90,1.00)	0.063
Age group			
< 60 year	60	1	
60–69 years	38	1.41 (1.27,1.56)	<0.001
70–79 years	31	1.76 (1.60,1.93)	<0.001
> = 80 year	27	1.94 (1.77,2.14)	<0.001
SIMD			
1. Most deprived	33	1	
2	32	0.98 (0.91,1.05)	0.533
3	33	0.98 (0.90,1.06)	0.618
4	33	0.96 (0.88,1.06)	0.445
5. least deprived	36	0.83 (0.75,0.91)	<0.001
ICD10_site			
C77	71	1	
C78	29	2.21 (1.86,2.63)	<0.001
C79	53	1.49 (1.25,1.79)	<0.001
C80	28	2.27 (1.91,2.71)	<0.001
Morphology			
Adenocarcinoma	48	1	
SCC	89	0.63 (0.52,0.76)	<0.001
NoS	26	1.53 (1.44,1.64)	<0.001
Other	84	0.61 (0.51,0.72)	<0.001
Type of admission			<0.001
Elective	75	1	
Emergency	30	1.88 (1.72,2.06)	<0.001
Specialty of admission			<0.001
Geriatric medicine	28	1	
Medicine	30	0.87 (0.79,0.95)	0.002
Surgery	40	0.66 (0.60,0.73)	<0.001
Other	44	0.52 (0.43,0.63)	<0.001

*SIMD* Scottish Index of Multiple Deprivation, *ICD10_site* International Classification of Diseases, 10^th^ Revision, anatomical site, *SCC* squamous cell carcinoma, *NoS* not otherwise specified, *HR* hazard ratio, C77 secondary and unspecified malignant neoplasm of lymph nodes, C78 secondary malignant neoplasm of respiratory and digestive organs, *C79* secondary malignant neoplasm of other sites, *C80* malignant neoplasm without specification of site

Increasing age was associated with poorer survival (P < 0.001). Median survival in the most deprived quintile (33 days) was significantly poorer than in the least deprived (36 days, P < 0.001) but was not significantly different from the other socio-economic quintiles. There was a significant association between ICD-10 site and survival (P <0.001); ICD-10 site C77 (Secondary and unspecified malignant neoplasm of lymph nodes of head, face and neck) had longest median survival (71 days) compared with C79 (secondary malignant neoplasm of other sites) at 53 days. Sites 78 and C80 (secondary malignant neoplasm of lung and malignant neoplasm without specification of site, respectively) had similar median survival at 29 & 28 days respectively. Morphology was also significantly associated with survival (*P* <0.001); SCC had the longest survival (median 89 days); NOS had the poorest survival (median 26 days). Emergency admissions had poorer survival than elective admissions, with median survivals of 30 and 75 days, respectively (*P* <0.001). Patients who were admitted to geriatric medicine wards had the poorest survival, (median 28 days) while admissions to “other” specialties had the longest median survival (44 days). There was no significant association between sex and survival.

Table 4 summarises a multivariate analysis of survival. The largest effect on survival was site, followed by morphology, type of admission, age, sex and speciality of admission. Deprivation was not significant once morphology, type of admission, age, sex and speciality were in the model.

**Table 4:** Multivariate analysis of survival of patients diagnosed with cancer of unknown primary during hospital admission, 1998–2011, West of Scotland, *N* = 5397

	HR	(95% confidence interval)	*P*
Morphology			
Adenocarcinoma		1	
SCC	0.89	(0.73, 1.07)	0.216
NoS	1.43	(1.34, 1.53)	<0.001
Other	0.66	(0.56, 0.79)	<0.001
ICD10_site			
C77		1	
C78	1.79	(1.50, 2.14)	<0.001
C79	1.10	(0.91, 1.32)	0.32
C80	1.79	(1.50, 2.14)	<0.001
Type of admission			
Elective		1	
Emergency	1.63	(1.49, 1.79)	<0.001
Age_group			
< 60 year		1	
60–69 years	1.32	(1.19, 1.47)	<0.001
70–79 years	1.47	(1.34, 1.62)	<0.001
> = 80 year	1.44	(1.30, 1.59)	<0.001
Gender			
Male		1	
Female	0.89	(0.84, 0.94)	<0.001
Specialty of admission			
Geriatric medicine		1	
Medicine	0.89	(0.82, 0.98)	0.017
Surgery	0.82	(0.75, 0.91)	<0.001
Other	0.78	(0.64, 0.95)	0.011

*ICD10_site* International Classification of Diseases, 10^th^ Revision, anatomical site, *SCC* squamous cell carcinoma, *HR* hazard ratio, C77 secondary and unspecified malignant neoplasm of lymph nodes, C78 secondary malignant neoplasm of respiratory and digestive organs, C79 secondary malignant neoplasm of other sites, *C80* malignant neoplasm without specification of site, *NoS* not otherwise specified

## Discussion

In a large population-based series of patients with Cancers of Unknown Primary, we found that the majority were diagnosed during a hospital admission, half of whom died during that admission. Survival after admission was short, being a median of 33 days and poorer in emergency compared to elective admissions. Median age was 75 years.

Survival in our study was less than the median 2–3 months reported by others [2–5, 10]. This may reflect that patients in our study were diagnosed during a hospital admission and may therefore have more advanced CUP or other morbidities requiring admission. We also found that a considerably smaller proportion of patients survived 5 years compared with others’ observations [2–5]. Long-term survivors are considered as belonging to a favourable prognostic subset, who respond to treatment for a primary cancer that CUP appears to mimic [6]. Research into treatment regimens is increasing for patients in both unfavourable and favourable prognostic subsets; the ability to identify those most likely to benefit from these treatments is important. Despite this, those in the unfavourable prognostic subset have limited treatment options, realistic aims being modest prolongation of life and symptom relief [9].

Younger patients who were admitted electively have the best survival from CUP. This is consistent with demographic prognostic indicators noted previously. This raises questions of whether physiological or pathological processes can explain the development of CUP; newer genetic profiling and imaging techniques are being used to try to clarify the underlying physiological and pathological processes in CUP development. Inequity regarding treatment by sex, age and deprivation is unlikely as currently there are no proven treatments for patients belonging to the unfavourable prognostic subset.

The increased risk of death in patients admitted to geriatric medicine in multivariate analysis despite adjustment for factors including age, sex, admission type and CUP site and morphology codes is interesting and the underlying reasons have not been determined. These patients may be more frail or have multiple comorbidities. It is unlikely that their increased risk of death is due to treatment because, as previously discussed, currently treatment options are limited and unlikely to significantly improve survival. We found that survival appeared better for ICD-10 C77 and “other” morphologies. It is probable that some lymph node metastases (C77) will represent regional rather than distant spread which may explain their better survival on average. Further research may be possible to better understand whether particular morphological “other” groups are associated with better survival but numbers may be small. We found that the morphology was coded as cancer “not otherwise specified” (NOS) in 67% of all CUP incidences. We anticipate that this was most commonly because biopsy and microscopic assessment (i.e. pathological confirmation) have not been undertaken, often because this would not influence patient management and/or because of the patient’s poor performance status. In Brewster’s previous study of CUP in the Scottish population between 2001 and 2010, 58% of CUP cases were recorded as not microscopically verified [7]. We anticipate that a proportion of incidences coded as cancer NOS will have shown a poorly differentiated tumour, preventing more detailed cancer typing and sub-typing.

Given the poor outcomes of patients admitted to hospital with CUP, is there scope for better primary and secondary prevention? Current understanding of the underlying biology and pathogenesis of CUP is incomplete [13]; therefore, the possibility of detecting CUP earlier is uncertain. Unlike other cancers, for example ovarian, rather than following a type 1 progression (from a pre-malignant to malignant lesion) it may be malignant from the start – that is, type 2 progression [6, 14]. Furthermore, the heterogeneity of CUP - its occurrence at any site as a variety of histological morphologies - does not lend it to a simple and acceptable method for early detection. Heterogeneity also results in a wide range of signs and symptoms, making it a poor candidate for a public health education campaign aiming to increase awareness of potential signs and symptoms of cancer and encourage primary care consultation. An issue with this health awareness approach in patients with CUP is that late presentation is often due to the lack of symptoms until advanced stages of the disease rather than failure to present to health services amongst symptomatic patients. There is evidence of CUP clustering in families where a sibling has experienced CUP, colorectal, hepatic, breast, ovarian, lung and renal cancers; suggesting a genetic basis to development of CUP, an area for future research, which may aid earlier detection and prevention of CUP [15].

Our study has strengths and weaknesses. Its strengths include the use of high quality cancer registry data and linkage to comprehensive hospital admission and death records, making it unlikely that significant numbers of patients have been excluded from our sample. The largest weakness – which is common to the study of CUP – is that it is based on a diagnosis of exclusion rather than confirmation and thus a product of the investigations that are deemed appropriate for any individual. Further research may be possible into investigations made prior to diagnosis using Scottish Morbidity Records (hospitalisation records), which include up to four paired Office of Population Censuses and Surveys version 4 operations and procedures codes. It would also be potentially possible to study prior and concurrent co-morbidities which may influence what investigations and/or treatments might be tolerated by patients, as well as outcome.

## Conclusions

Cancers of Unknown Primary have poor outcomes and the opportunities to better identify and manage such patients may be limited because of patients’ short survival after hospital admission. Supportive and palliative care may therefore be key contributions that specialist CUP teams can offer patients. For those patients who present with more favourable disease, however, early effective treatment is a priority. Further research on investigations prior to diagnosis may help to inform more efficient diagnostic pathways that could benefit patients in either prognostic group.

## Abbreviations

CI: Confidence interval
CUP: Cancers of unknown primary
ESMO: European society for medical Oncology
HR: Hazard ratio
ICD-10: International classification of diseases and related health problems, 10^th^ revision
ICD-O: International classification of diseases for oncology
NICE: National institute for health and care excellence
NOS: Not otherwise specified
SCC: Squamous cell carcinoma
SIMD: Scottish index of multiple deprivation
SMR: Scottish morbidity record
UK: United kingdom

## References

[1] National Institute for Health and Clinical Excellence: Metastic malignant disease of unknown primary origin: Diagnosis and management of metastatic malignant disease of unknown primary origin. London: NICE; 2010. 31886996

[2] Shu X, Sundquist K, Sundquist J, Hemminki K (2012). Time trends in incidence, causes of death, and survival of cancer of unknown primary in Sweden. Eur J Cancer Prev.

[3] Luke C, Koczwara B, Karapetis C, Pittman K, Price T, Kotasek D, Beckmann K, Brown MP, Roder D (2008). Exploring the epidemiological characteristics of cancers of unknown primary site in an Australian population: implications for research and clinical care. Aust N Z J Public Health.

[4] Urban D, Rao A, Bressel M, Lawrence YR, Mileshkin L (2013). Cancer of unknown primary: a population-based analysis of temporal change and socioeconomic disparities. Br J Cancer.

[5] Randen M, Rutqvist LE, Johansson H (2009). Cancer patients without a known primary: incidence and survival trends in Sweden 1960–2007. Acta Oncol.

[6] Pavlidis N, Pentheroudakis G (2012). Cancer of unknown primary site. Lancet.

[7] Brewster DH, Lang J, Bhatti LA, Thomson CS, Oien KA (2014). Descriptive epidemiology of cancer of unknown primary site in Scotland, 1961–2010. Cancer Epidemiol.

[8] Cancer Research UK and National Cancer Intelligence Network (2013). Cancer of unknown primary NCIN and CR-UK data briefing.

[9] Fizazi K, Greco FA, Pavlidis N, Pentheroudakis G, Group EGW (2011). Cancers of unknown primary site: ESMO clinical practice guidelines for diagnosis, treatment and follow-up. Ann Oncol.

[10] National Cancer Intelligence Network (2014). Routes to diagnosis: cancer of unknown primary – NCIN data briefing.

[11] Fritz A, Percy C, Jack A. International Classification of Diseases for Oncology. 3rd edn. Geneva: World Health Organisation; 2000.

[12] Scottish Government. http://www.scotland.gov.uk/Topics/Statistics/SIMD/BackgroundMethodology. Accessed 27 Jan 2017.

[13] Greco FA (2012). Cancer of unknown primary site: evolving understanding and management of patients. Clin Adv Hematol Oncol.

[14] Pavlidis N, Pentheroudakis G (2010). Cancer of unknown primary site: 20 questions to be answered. Ann Oncol.

[15] Hemminki K, Ji J, Sundquist J, Shu X (2011). Familial risks in cancer of unknown primary: tracking the primary sites. J Clin Oncol.

